# A Rare Case of a Pelvic Solitary Fibrous Tumor

**DOI:** 10.7759/cureus.23686

**Published:** 2022-03-31

**Authors:** Claudia Gordillo, Mary Raymond, Ryan De Melo

**Affiliations:** 1 Radiology, Aventura Hospital and Medical Center, Aventura, USA

**Keywords:** images, radiology, pelvis, extra-pleural, solitary fibrous tumor

## Abstract

Solitary fibrous tumors (SFTs) are rare neoplasms commonly arising from the pleura. Multiple extra-pleura occurrences have been established by the literature. CT scan and MRI with IV contrast serve as the steppingstone for early detection and diagnosis, and to evaluate disease burden. Surgical resection is most of the time curative. SFTs carry a malignant potential and may recur or metastasize thus long-term follow-up is of utmost importance for patients diagnosed with this tumor. We discuss the case of a 47-year-old male patient who presented with urinary retention, caused by a large pelvic tumor pathologically proven to be a solitary fibrous tumor.

## Introduction

Solitary fibrous tumors (SFTs) are rare mesenchymal tumors mainly originating from the pleura [1]. Multiple extra-thoracic locations have been documented such as the abdominopelvic region, thymus, liver, thyroid, nasal cavity, and peritoneum [1]. It has been reported in the literature that approximately 30% of cases occur in the abdominopelvic cavity [1]. The majority of solitary fibrous tumors are benign, with up to 20% of cases reported to be malignant [2]. Imaging plays a pivotal role in helping establish the diagnosis and evaluating the extent of the disease [3]. Local recurrence and distant metastasis may be expected with malignant SFTs but have also been documented in histologically benign SFTs [4]. Complete surgical resection is the treatment of choice with neoadjuvant therapy demonstrating varying success [1].

## Case presentation

**Figure 1:**
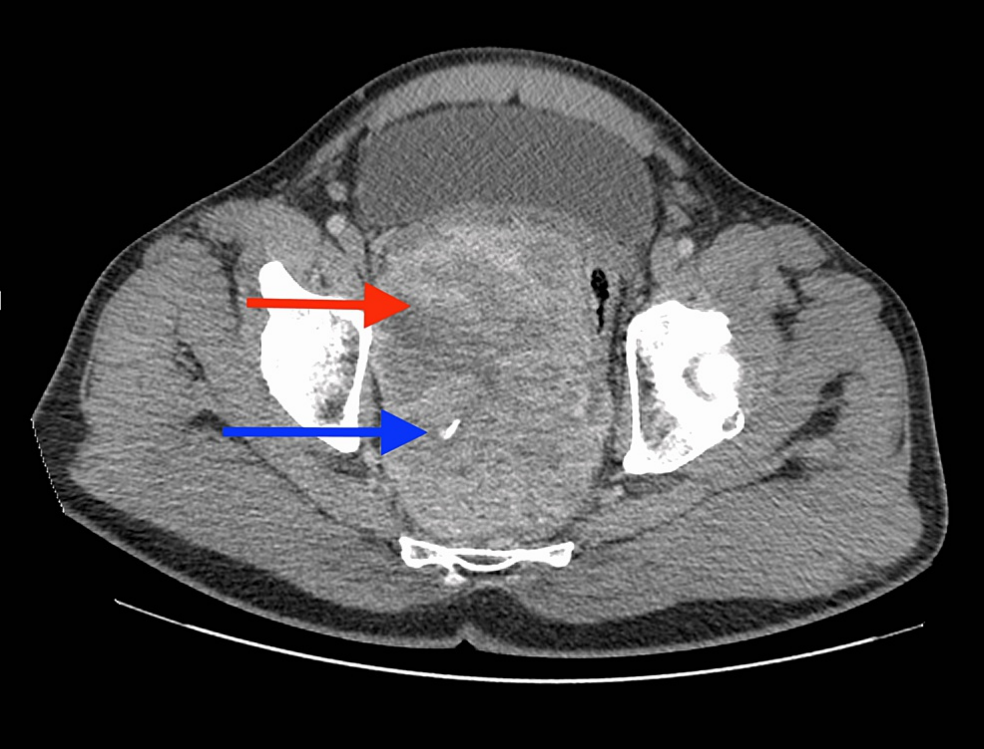
Axial contrast-enhanced images of the pelvis demonstrating a large heterogeneous mass (red arrow) with calcifications (blue arrow)

**Figure 2:**
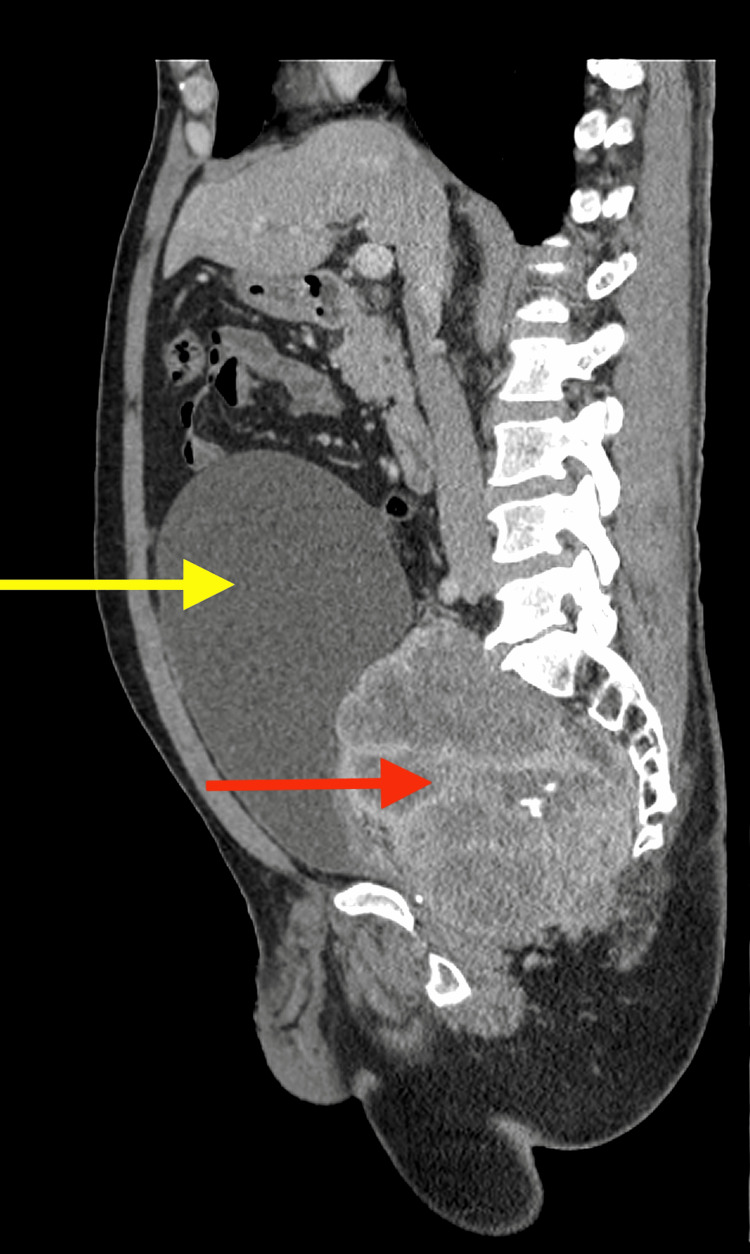
Sagittal contrast images show the same mass (red arrow) exerting a mass effect and displacing the urinary bladder superiorly (yellow arrow)

**Figure 3:**
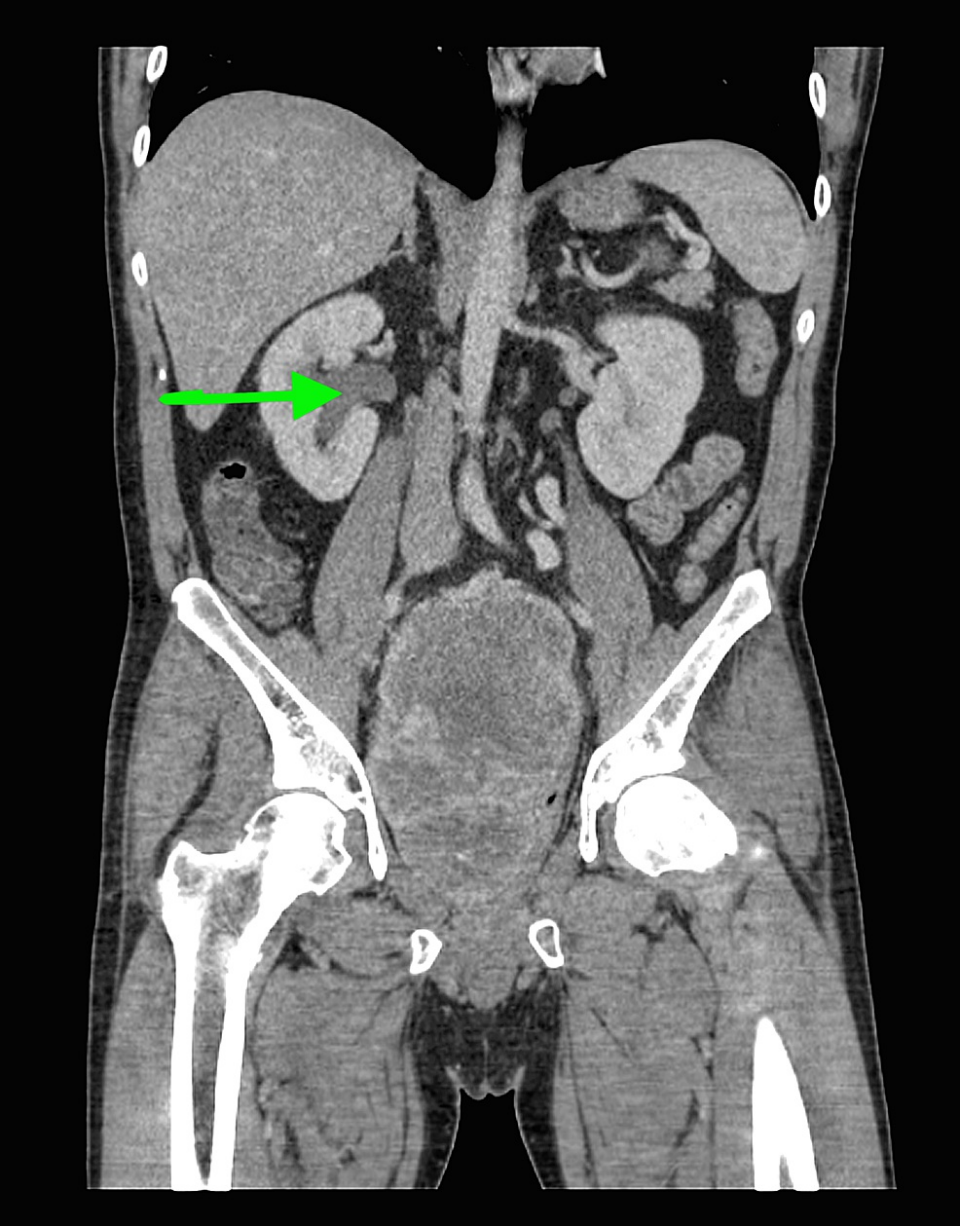
Coronal image illustrates right-sided hydronephrosis (green arrow) caused by the pelvic mass

The patient is a 47-year-old Hispanic male with no reported past medical history who presented to the emergency room complaining of difficulty urinating. The patient did not recall the exact onset of his symptoms but stated they had progressively worsened over the course of the past year. CT scan of the abdomen and pelvis with intravenous iodinated contrast showed a 7.5 x 9.7 x 15.0 cm multinodular, heterogeneous pelvic mass with calcifications causing mild to moderate hydronephrosis and urinary bladder distention (Figure 1, Figure 2, and Figure 3).

**Figure 4:**
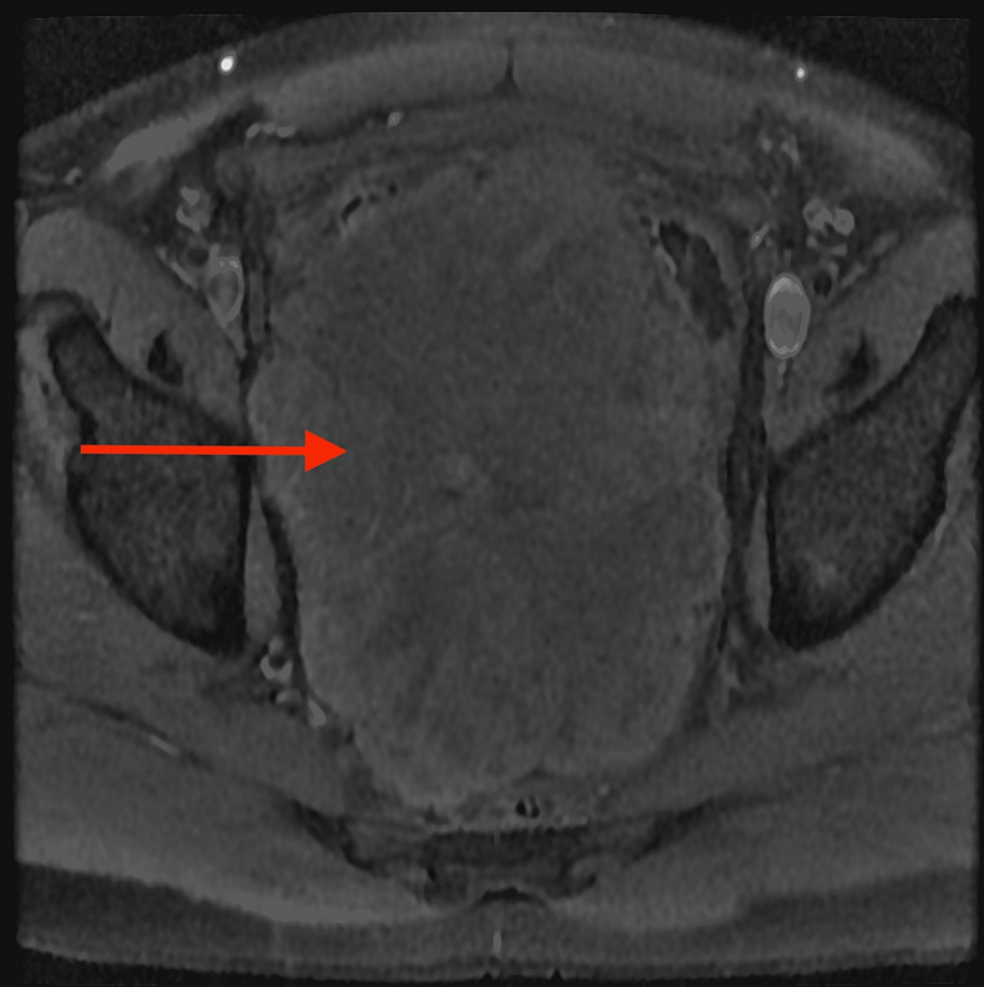
Axial MRI T1 pre-contrast image demonstrates a large lobulated mass (red arrow)

**Figure 5:**
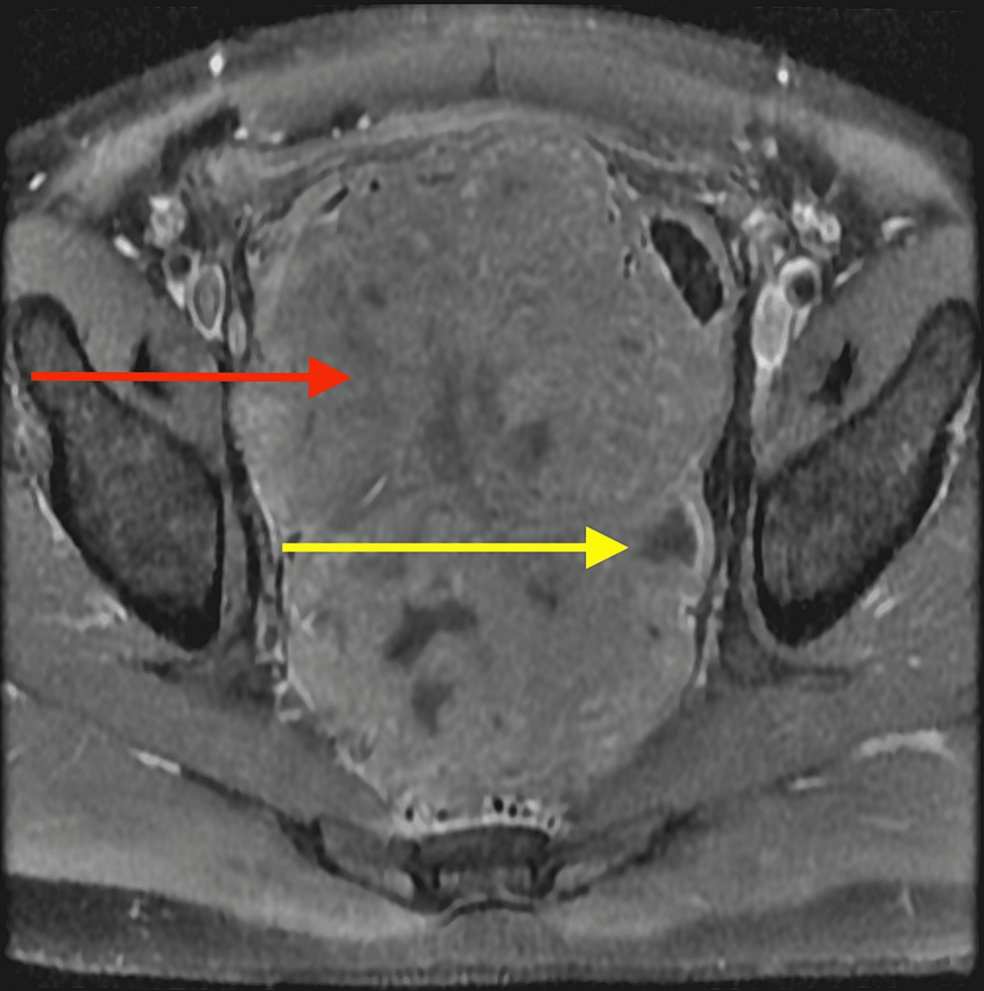
Axial MRI T1 post-contrast sequence shows an avidly enhancing heterogeneous mass (red arrow) with multiple areas of necrosis (yellow arrow)

**Figure 6:**
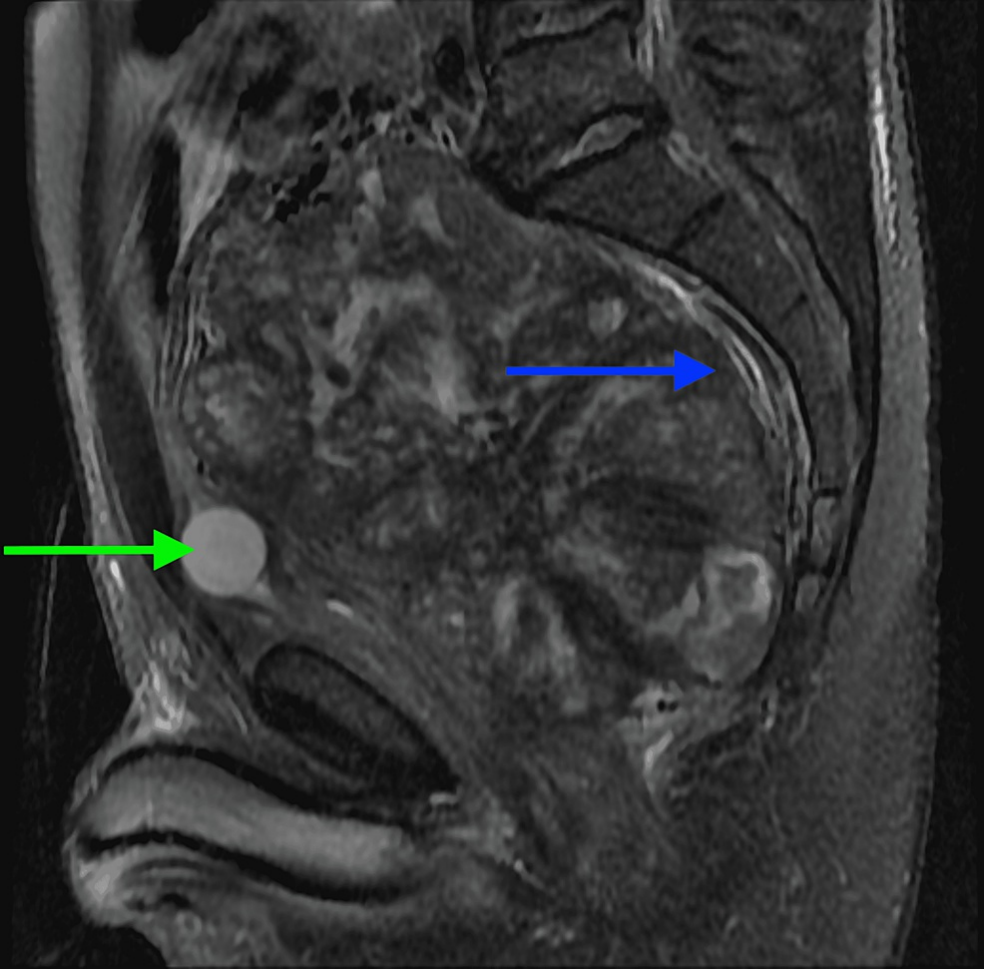
Sagittal T2 sequence shows the same pelvic mass exerting a severe mass effect on the rectum (blue arrow). The urinary bladder is decompressed with a Foley catheter in place (green arrow)

**Figure 7:**
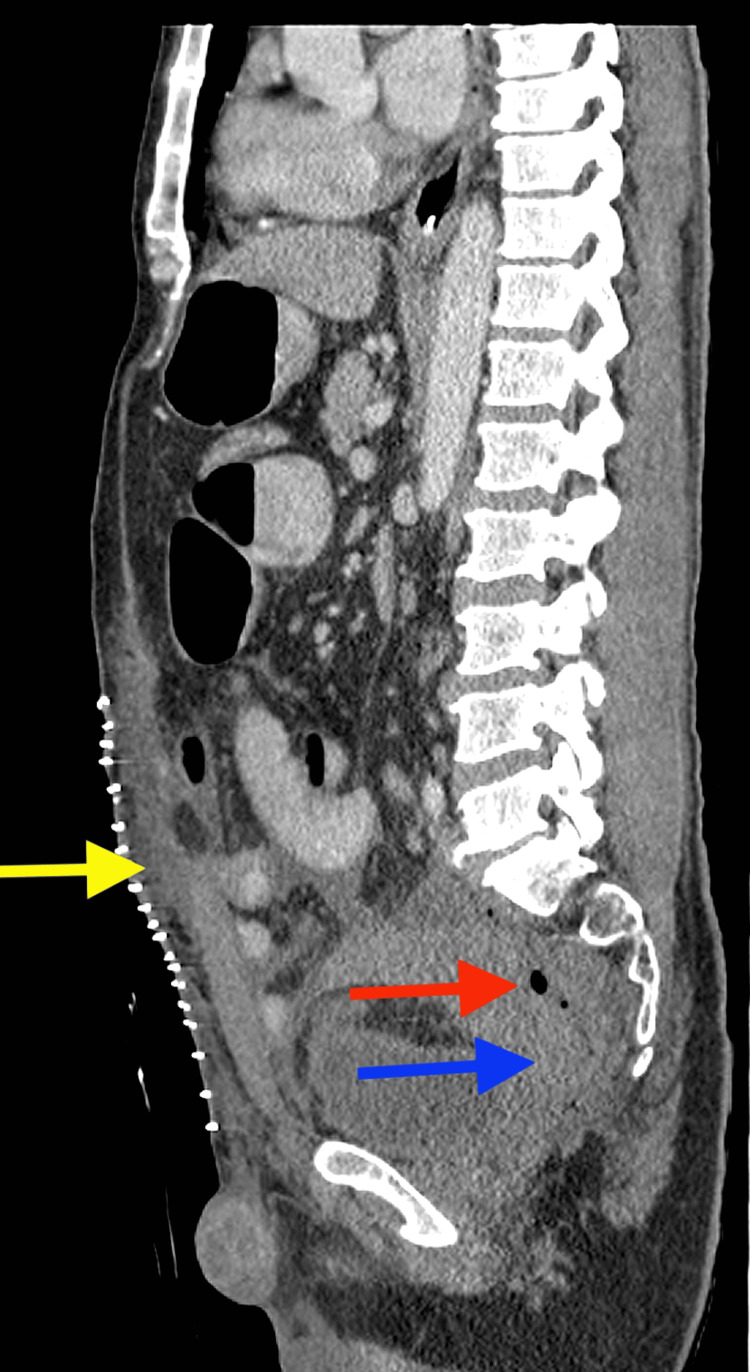
Sagittal contrast-enhanced images of the abdomen and pelvis show postoperative changes within the anterior abdominal wall (yellow arrow) and post-surgical changes within the pelvis, including air (red arrow), fluid, and blood products (blue arrow)

A Foley catheter was placed in the ER with mild symptomatic relief. Follow-up MRI of the pelvis with IV contrast demonstrated a heterogeneously enhancing pelvic mass measuring approximately 10.3 x 14.0 x 16.0 cm and exerting a significant mass effect on the rectum with multiple areas of necrosis (Figure 4, Figure 5, and Figure 6). No metastatic lesions or adenopathy were identified by imaging. Pathology from the transrectal biopsy specimen yielded a solitary fibrous tumor positive for BCL2 and CD34 and negative for ALK1, S100, and desmin. The patient subsequently underwent exploratory laparotomy with radical resection of the pelvic tumor, abdominal-perineal resection of the rectum, and creation of end colostomy in the left lower quadrant since the tumor was found to involve the rectum intraoperatively. Surgical pathology was consistent with a solitary fibrous tumor, moderate risk, involving the rectal wall and perirectal adipose tissue, and positive for CD34 and negative for CKIT. The proximal and distal resection margins were free of tumor and lymphovascular and perineural invasion was not identified. One-day postoperative CT scan of the abdomen and pelvis with IV contrast demonstrated post-surgical changes within the anterior abdominal wall with expected fluid, air, and blood products in the surgical bed (Figure 7). Upon discharge, the patient agreed to schedule an outpatient follow-up with Hematology-Oncology to establish a care plan. 

## Discussion

SFTs are rare mesenchymal neoplasms often encountered in the pleura, accounting for less than 5% of all pleural tumors [5]. It has been documented in the literature that SFTs can essentially originate in any site of the body with extra-pleural tumors occurring more commonly than pleural SFTs [3]. Solitary fibrous tumors often present during the fifth and sixth decade of life without significant sex predilection [6]. Patients with extra-pleural SFTs are usually symptomatic at the time of presentation [7]. Symptoms are secondary to mass effects and include abdominal pain/fullness, change in bowel habits, and lower urinary tract complaints [7]. Hypoglycemia has also been reported as a rare, associated symptom that is thought to be secondary to the production of an insulin-like growth factor in some tumors [6].

Histologically, SFTs are composed of proliferative spindle cells in a background of dense collagenous stroma and numerous thin-walled blood vessels [8]. Immunohistochemistry is critical in differentiating SFTs from other spindle cell neoplasms. Immunopositivity for CD34 is key in diagnosing SFTs with most tumors also positive for CD99 and Bcl-2 as well as negative staining for S100 [8]. Malignant SFTs tend to lose reactivity to CD34, overexpress p53, S-100, and Ki-67, and exhibit a higher degree of necrosis [4].

The radiological appearance of SFTs is variable and nonspecific [5]. A CT scan is the modality of choice for initial imaging, often demonstrating a well-circumscribed hypervascular lesion that exerts a mass effect on neighboring organs with central areas of necrosis and rarely calcifications [3]. On MRI, SFTs usually exhibit an intermediate signal on T1-weighted sequences, heterogeneously low signal on T2-weighted images, and intense enhancement after IV contrast administration [3]. The degree of contrast enhancement on both CT and MRI imaging varies from moderate to marked with an early intense arterial enhancement of the hypervascular areas, moderate enhancement of the hypercellular areas, and no enhancement of the areas of myxoid degeneration and necrosis [8]. On ultrasound, SFTs typically present as hypoechoic masses but may occasionally appear heterogeneous secondary to the necrotic and degenerative changes [6]. Despite the highly vascular nature of these tumors, flow is not always seen on Doppler imaging [6]. Benign SFTs exhibit low-grade fluorodeoxyglucose PET activity while malignant SFTs tend to be hypermetabolic with increased radiotracer uptake [6].

Surgical resection is the treatment of choice for SFTs and is curative in most cases of benign SFTs [7]. Embolization of feeding arteries may be used to shrink tumors prior to surgical resection and help prevent excessive intraoperative bleeding [1]. It has been documented in the literature that tumors greater than 10 cm have a significantly higher risk of local disease recurrence and metastasis, particularly those demonstrating histologically malignant components [7]. Invaded surgical margins are the highest predictor of local recurrence thus complete surgical resection with negative margins should be ensured [4]. There are varying reports on the success of neoadjuvant therapy for SFTs [1], thus it may be offered to patients on a case-to-case basis. Long-term follow-up is of utmost importance for patients with SFTs given the potential for recurrence and metastasis regardless of anatomic location [4].

## Conclusions

Solitary fibrous tumors of the pelvis are rare extra-pleural neoplasms with malignant potential. Imaging features help establish the diagnosis and assess the disease burden. On CT and MRI images, which are often the initial test of choice, these tumors appear as large, heterogeneously enhancing masses with areas of necrosis. Complete surgical resection with negative margins is the treatment of choice for SFTs. It has been reported that embolization of feeding arteries prior to resection may improve surgical outcomes and reduce complications. Given the potential for malignancy and the possibility of disease recurrence and metastasis, long-term follow-up is crucial for patients diagnosed with SFTs of the pelvis.
